# Identification of Protein Complex Associated with LYT1 of *Trypanosoma cruzi*

**DOI:** 10.1155/2013/493525

**Published:** 2013-03-17

**Authors:** C. Lugo-Caballero, G. Ballesteros-Rodea, S. Martínez-Calvillo, Rebeca Manning-Cela

**Affiliations:** ^1^Departamento de Biomedicina Molecular, Centro de Investigación y Estudios Avanzados del IPN, Avenida Instituto Politécnico Nacional No. 2508, Col. San Pedro Zacatenco, 07360 Gustavo A. Madero, DF, Mexico; ^2^UBIMED, Facultad de Estudios Superiores Iztacala, Universidad Nacional Autónoma de México, Avenida De los Barrios 1, Col. Los Reyes Iztacala, 54090 Tlalnepantla, MEX, Mexico

## Abstract

To carry out the intracellular phase of its life cycle, *Trypanosoma cruzi* must infect a host cell. Although a few molecules have been reported to participate in this process, one known protein is LYT1, which promotes lysis under acidic conditions and is involved in parasite infection and development. Alternative transcripts from a single *LYT1* gene generate two proteins with differential functions and compartmentalization. Single-gene products targeted to more than one location can interact with disparate proteins that might affect their function and targeting properties. The aim of this work was to study the LYT1 interaction map using coimmunoprecipitation assays with transgenic parasites expressing LYT1 products fused to GFP. We detected several proteins of sizes from 8 to 150 kDa that bind to LYT1 with different binding strengths. By MS-MS analysis, we identified proteins involved in parasite infectivity (trans-sialidase), development (kDSPs and histones H2A and H2B), and motility and protein traffic (dynein and **α**- and **β**-tubulin), as well as protein-protein interactions (TPR-protein and kDSPs) and several hypothetical proteins. Our approach led us to identify the LYT1 interaction profile, thereby providing insights into the molecular mechanisms that contribute to parasite stage development and pathogenesis of *T. cruzi* infection.

## 1. Introduction

American trypanosomiasis is a disease that is caused by *Trypanosoma cruzi*, an obligate intracellular parasite that infects a variety of mammalian host cells. This disease is endemic in Latin America, where it affects approximately 18 million people, and more than 100 million people are at risk of infection [1]. *T. cruzi *undergoes a complex biphasic life cycle that alternates between two developmental stages in the reduviid beetle vector (i.e., epimastigotes and metacyclic trypomastigotes) and two developmental stages in the mammalian host (i.e., amastigotes and blood trypomastigotes). In the beetle, the flagellated epimastigote proliferates in the midgut before differentiating into the nondividing but infectious metacyclic trypomastigote, which is found in the vector's hindgut. The parasite infects host cells after its introduction into mammalian blood, differentiates into the amastigote, and initiates replication in the cytosol of the infected cell. Ultimately, the amastigotes develop into nondividing bloodstream trypomastigotes, which can either initiate another round of infection or be ingested by the reduviid vector during a blood meal. The life cycle is completed upon the development of epimastigotes from bloodstream trypomastigotes.

 Although the infection process of *T. cruzi* was described many years ago, the molecular mechanisms involved remain poorly understood. The parasite infects diverse professional and nonprofessional phagocytes by a process that appears to involve several discrete steps, beginning with the attachment of the parasite to the host cell and followed by its internalization through a parasitophorous vacuole, from which it escapes to multiply freely in the cytosol. Subsequently, it differentiates into the bloodstream trypomastigote form and is ultimately liberated from the host cell. Although many proteins are undoubtedly important for *T. cruzi* infection, surprisingly few have been identified experimentally. However, one such protein is LYT1, which is a lytic protein that plays a critical role in the parasite infection and stage transition processes [2]. *LYT1* is a single-copy gene that encodes three distinct *LYT1* mRNAs through alternative trans-splicing of the primary transcript, which is differentially regulated during the parasite life cycle. Two transcripts encode full-length LYT1 proteins that contain an N-terminal signal sequence and a nuclear localization sequence, and the third transcript encodes a truncated LYT1 protein lacking the signal sequence and only containing the nuclear localization sequence [3]. *LYT1*-deficient parasites are infection deficient, display accelerated *in vitro* development, and have diminished hemolytic activity in acidic conditions [2]. The differential reconstitution of the two *LYT1* products in null parasites showed that the full form of the protein is localized to the plasma membrane and reverts the infection deficiency phenotype, while the truncated form of the protein is localized in the mitochondrial kinetoflagelar zone and reverts the accelerated *in vitro* stage differentiation phenotype [4]. The differential localization of the full and truncated forms of LYT1 was later confirmed using transgenic parasites that express an exogenous copy of LYT1 fused to EGFP. Furthermore, these studies also revealed that both forms of the LYT1 protein are localized in the nucleus and kinetoplast zone [5].

 It is well known that single eukaryotic genes can give rise to proteins that are localized to several subcellular localizations, an event referred to as dual targeting, dual localization, or dual distribution. This event occurs through one of several routes that are based on more than one gene, more than one mRNA from a single gene, or more than one translation initiation on a single mRNA, which can result in different translation products that differ by the presence or absence of specific targeting signals [6]. Repetitious forms of the same protein with identical or nearly identical sequences that are distinctly localized in the cell have been recently called “echoforms” to distinguish them from “isoproteins,” which are proteins with the same activity but different amino acid sequences [6]. Proteins that harbor one signal, two separate signals or an overlapping ambiguous signal may also undergo dual distribution in the cell. The mechanism of this dual targeting is driven by the competition or promiscuity of various molecular events that involve protein folding, posttranslational modification, and protein-protein interaction [7].

 Subcellular compartments and organelles contain specific proteins that determine their structure and function [7]. Most proteins carry out their functions within a complex network of interactions in which a single component can affect a wide range of other components [8]. If two proteins interact with one another, they usually participate in the same, or related, cellular pathway(s), and clues to the function of a protein can be obtained by determining its interactions with another protein of known function [8, 9]. Therefore, understanding how proteins interact is a significant area of current research.

 The dual localization of LYT1 exposes this molecule to different microenvironments and the possibility of interactions with other proteins that could promote different functionality. For this reason, in this work, we began to unravel the LYT1 interaction profile by coimmunoprecipitation assays using stably transfected parasites expressing an exogenous LYT1 protein fused to the enhanced green fluorescent protein (EGFP). The advantage to this *in vivo* approach is that it can be carried out while maintaining intracellular conditions, thereby enabling a better analysis of the LYT1 interaction profile and the possible influence that this could have on the different pathways in which LYT1 is involved.

## 2. Materials and Methods

### 2.1. Parasites

Epimastigotes from wild-type (WT) and LYT1s+n-EGFP transgenic *T. cruzi *Cl-Brener strains were maintained in liver infusion tryptose medium (LIT) containing 10% FBS, 0.5% penicillin (10,000 IU)/streptomycin (10 *μ*g), and 0.5% of hemin (5 mg/mL) at 28°C [2]. Mid-log-phase cultures were used in all experiments.

### 2.2. Construction of pTREXn-LYT1s+n-EGFP and pTREXn-EGFP

**Figure 1:**
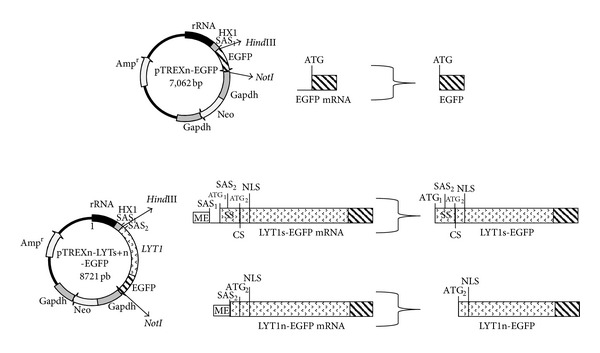
Diagrams representing the pTREXn-EGFP and pTREXn-LYT1s+n-EGFP constructs, transcripts, and protein products (not to scale). The presence and relative position of trans-splicing acceptor sites (SAS_1_ and SAS_2_), miniexon (ME), translations initiation sites (ATG1 and ATG_2_), signal sequence (SS), nuclear localization sequence (NLS), and cleavage site (CS) are shown for the mRNA (EGFP mRNA, LYT1s-EGFP mRNA, and LYT1n-EGFP mRNA) and corresponding proteins (EGFP, LYT1s-EGFP, and LYT1-EGFP). Striped boxes represent EGFP sequences. Arrowheads pointing to filled boxes represent LYT1 sequences. Restriction sites are given. Details of the plasmid construction are described in “Section 2.”

The *LYT1s+n* sequence was amplified by PCR with 1 unit of Herculase Taq DNA polymerase (Cat. number 600262-51/Stratagene), the buffer provided by the manufacturer, dNTPs, the *LYT1b* allele DNA as a template (GenBank AF320626) [2], and the specific oligonucleotides LYT11 S [5′-GCG GAA TTC ATG CGG AAG AAA GCC GCA GC-3′/nucleotide (nt) 1–20 of the *LYT1* coding sequence (GenBank AF320626) downstream of the *Eco*RI (nt 4–9) site] and LYT17 AS [5′-GGG GTA CCC CAT CAG CTG CCA GCA TGT TTT C-3′/complementary to nt 1631–1656 of the *LYT1* coding sequence (GenBank AF320626) downstream of the *Kpn*I (nt 3–6) site]. The PCR conditions used were as follows: the enzyme was added to a first cycle of 1 min at 95°C, followed by 30 cycles of 1 min at 95°C, 1 min at 60°C, 3 min at 72°C, and a final cycle of 7 min at 72°C. The PCR product was digested with *Eco*RI and *Kpn*I, purified by gel electrophoresis, and cloned in the commercial vector pEGFP-N1 (Clontech), which had been previously digested with the corresponding restriction enzymes. The resultant plasmid was digested with *Eco*RI and *Kpn*I and gel purified to obtain the chimera *LYT1s+n-EGFP,* which was subsequently subcloned in pTREXn [10] in the corresponding restriction enzymes sites. The *EGFP* sequence was obtained by the digestion of pEGFP-N1 (Clontech) with *Eco*RI and *Kpn*I and, after gel purification, was subcloned in pTREXn. The resultant plasmids pTREXn-LYT1s+n-EGFP and pTREXn-EGFP (Figure 1) were used for transfection experiments after verifying the correct fusion of *LYT1s+n* to *EGFP* and cloning by sequence analysis.

### 2.3. Generation of LYT1-Containing Stably Transfected Parasites

Mid-log-phase WT epimastigotes (3 × 10^8^) from the *T. cruzi* CL-Brener strain, resuspended in cold LYT medium, were transfected by electroporation (BTX ECM 830) with 100 *μ*g of cesium chloride and purified pTREXn-LYT1s+n-EGFP or pTREXn-EGFP plasmid DNA at 300 volts for 12 ms in 2 mm BTX electroporation cuvettes. After electroporation, the transfected parasites were maintained for 5 min at 4°C and then transferred to fresh complemented LIT medium and incubated at 28°C. After 48 hr, the parasites were exposed to antibiotic selection with 500 g/mL of G418 (Cat. number 10131-035/GIBCO). Once antibiotic-resistant growth cultures were established, fluorescent clonal derivatives were isolated from each population of stably transfected parasites by flow cytometry (FACSVantage, Becton, Dickinson).

### 2.4. Total Protein Extraction

The parasites (1 × 10^8^) were washed three times with PBS pH 7.2 and then lysed with 500 *μ*L of lysis buffer (50 mM Tris-HCl, pH 7.8, 1% NP40, 5 mM EDTA, 1% SDS, 100 mM ZnCl_2_, and 1x complete proteinase inhibitor from Roche) at 4°C for 20 min. After two freeze-thaw cycles, the solubilized proteins were quantified by Lowry's technique (Cat. number 500-0114/DC Protein Assay Bio-Rad).

### 2.5. Western Blotting and Dot Blotting

The same amount of protein (100 *μ*g) was boiled with 1x sample buffer (1% SDS, 10% glycerol, 0.001% pironine, 0.06 M Tris-OH pH 6.8) and loaded into SDS-discontinuous polyacrylamide gel for electrophoresis (PAGE). The proteins were electrotransferred to nitrocellulose membranes (Bio Rad Laboratories), and the quantity of transferred proteins was verified by staining with ponceau/1% acetic acid. The destained membrane was blocked with 6% nonfat milk in 1x PBS and incubated with a 1 : 500 dilution in 6% non-fat milk/1x PBS of the antibody against EGFP (Santa Cruz Biotechnology, Inc./Cat. number SC-9996/monoclonal antibody obtained in mouse) overnight at 4°C. After three washes with 1x PBS, 0.05% tween-20 in 1x PBS, and 1x PBS for 10 min each, the secondary antibody HRP-conjugated anti-mouse IgG (H+L) (Santa Cruz Biotechnology, Inc./Cat. number SC-2380) was added at 1 : 5000 dilution in 6% non-fat milk/1x PBS to detect primary antibodies, for 1 hr at room temperature. The membranes were washed again as indicated before, and positive bands were visualized by chemiluminescence (Cat. number RPN2106 Amersham ECL). For dot blotting, the same amount of protein (10 *μ*g) was directly dripped over nitrocellulose membranes using a mini-fold Bio-Dot (Bio-Rad) and processed as western blots. Antibodies against EGFP (1 : 500 dilution) [Santa Cruz Biotechnology, Inc./Cat. No. SC-9996/monoclonal antibody obtained in mouse] and against the total protein of *T. cruzi* (1 : 1500 dilution) (polyclonal antibody obtained from mice in this work) were used as primary antibodies. The secondary antibody *α*-mouse IgG (H+L) conjugated to peroxidase [ZYMED LABORATORIES number cat. 81-6520/polyclonal antibody obtained in goat] was used to detect positive bands using 4-chloro-1-naphthol as the peroxidase substrate (Sigma/Cat. number C57804).

### 2.6. Coimmunoprecipitation Assay

These assays were performed using the Pro-Found coimmunoprecipitation kit (PIERCE 23600) exactly as indicated by the manufacturer. The antibody against EGFP (25 *μ*g) (Santa Cruz Biotechnology, Inc./Cat. No. SC-9996/monoclonal antibody obtained in mouse) was used for all the assays. The antibody against GST tag [ZYMED 13-6700] was used as an unrelated antibody control. To perform the coimmunoprecipitation, 50 *μ*g of total protein was diluted in coupling buffer to reach a final volume of 400 *μ*L, and this mixture was added to the corresponding columns, which were incubated for 8 hr at 4°C using an orbital mixer. Then, the columns were washed six times with 400 *μ*L of coupling buffer, and every wash fraction was collected and analyzed by SDS-PAGE to ensure the absence of protein. Finally, the columns were washed three times with 500 *μ*L of elution buffer to ensure the maximum recuperation of eluted proteins. All assay controls suggested by the manufacturer were simultaneously processed. The eluted fractions and wash fractions were concentrated using MICROCON columns (Millipore 42404 YM3). All the gels containing these controls and samples were silver stained using the kit SILVER-QUEST (Invitrogen LC6070). The stained gels were photographed with a Kodak DS290 digital camera under clear light and analyzed with the Kodak 1D version 3.5.4 software to determine the molecular weight (MW) of each band using the precision plus protein standards (BioRad 161-0374) as MW references. Western blot analysis was performed to verify the samples' integrity.

### 2.7. Mass Spectrometry Analysis

After 12% sample SDS-PAGE gel separation and silver staining, the protein bands were sent for analysis by MS-MS (Q-TOF) tandem mass spectrometry using an ESIA coupled to a Quadra pole ion trap TANDEM analyzer. Proteins were identified by MASCOT [11] and NCBI-*Blast* [12] software. For putative proteins *ExPASy-Prosite *[13]*, ExPASy-ProtParam *[14],* pSORT* [15], and* SMART *[16], software analyses were performed.

## 3. Results

### 3.1. Cloning and LYT1s+n-EGFP Stably Transfected Parasite Generation

**Figure 2:**
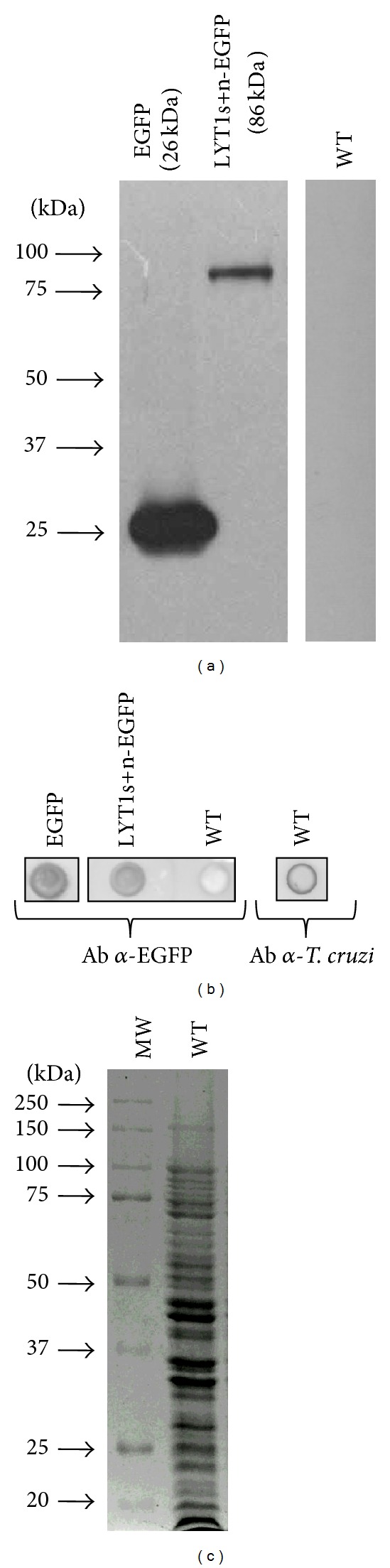
Analysis of EGFP and LYT1s+n-EGFP chimeric proteins expression. (a) Protein extracts of WT parasites and EGFP and LYT1s+n-EGFP transgenic parasites were run on polyacrylamide gels under denaturing conditions and transferred, and the exogenous proteins were revealed using an *α*-EGFP antibody. (b) Protein extracts of WT parasites and EGFP and LYT1s+n-EGFP transgenic parasites were dotted under native conditions and revealed using an *α*-EGFP or *α*-*T*. *cruzi* antibody. (c) Protein extract of WT parasites analyzed by SDS-PAGE and Coomassie-blue stain.

To identify the proteins that could bind to LYT1, we obtained transgenic parasites expressing LYT1 fused to EGFP. The pTREXn-LYT1s+n-EGFP construct was designed to express the full length and the truncated forms of LYT1 fused to EGFP (Figure 1). This plasmid contains two 3′ AG splice acceptor sites (SAS), one from the HXI vector sequence (SAS_1_) [10] and the other from +10 3′ AG of the LYT1 coding sequence (SAS_2_), and two ATG (+1/ATG_1_ and +85/ATG_2_) positions [3]; therefore, both the full-length (LYT1s-EGFP) and the truncated (LYT1n-EGFP) LYT1 proteins fused to EGFP are produced. The construct pTREXn-EGFP was used to express the *EGFP* sequence as a control. To evaluate the presence of EGFP and the LYT1s-EGFP and LYT1n-EGFP chimeras, the constructs described above were transfected into WT epimastigotes of the CL-Brener strain to generate the EGFP and LYT1s+n *T. cruzi* stable lines. To characterize the expression of exogenous EGFP and the LYT1s-EGFP and LYT1n-EGFP protein chimeras, western blot analysis of transfected parasites was performed. As shown in Figure 2(a), the monoclonal antibody against EGFP recognized the EGFP protein and the LYT1s-EGFP and LYT1n-EGFP protein chimeras in transfected parasites. Because the molecular weights of uncleaved (86 kDa) or cleaved (83 kDa) LYT1s-EGFP, and LYT1n-EGFP (83 kDa) are very close, the gel did not resolve separate bands; therefore, a single band was detected in parasites transfected with pTREXn-LYT1s+n-EGFP. Experiments using 6% SDS-PAGE showed the same results (data not shown). As expected, a band of 26 kDa corresponding to the EGFP protein was detected in parasites transfected with pTREXn-EGFP, and no EGFP was observed in WT parasites, demonstrating the specificity of the antibody.

 These results indicate that the stable lines expressed the exogenous sequences and demonstrate the successful generation of EGFP and LYT1s+n stably transfected parasite lines.

### 3.2. Recognition of the Recombinant Protein under Nondenaturing Conditions

Because the coimmunoprecipitation assay would be performed in nondenaturing conditions (Section 3.3), it was necessary to determine whether the antibody against EGFP was also able to recognize the EGFP, LYT1s-EGFP and LYT1n-EGFP exogenous proteins under these conditions. For this determination, a dot-blot assay was performed using total protein extracts of each line of transfected parasites under nondenaturing conditions as described in Section 2. As shown in Figure 2(b), the antibody was able to recognize EGFP and the LYT1s-EGFP and LYT1n-EGFP fusion proteins, indicating that the antibody could be used for the coimmunoprecipitation assays. No signal was observed in extracts from non-transfected parasites, demonstrating the specificity of the antibody (Figure 2(b)). The presence of the total protein extract of non-transfected parasites was confirmed using an antibody against the total protein of WT epimastigotes (Figure 2(b)) and its integrity by SDS-PAGE and Coomassie-blue staining (Figure 2(c)).

### 3.3. Identification of Proteins Associated with LYT1

**Figure 3:**
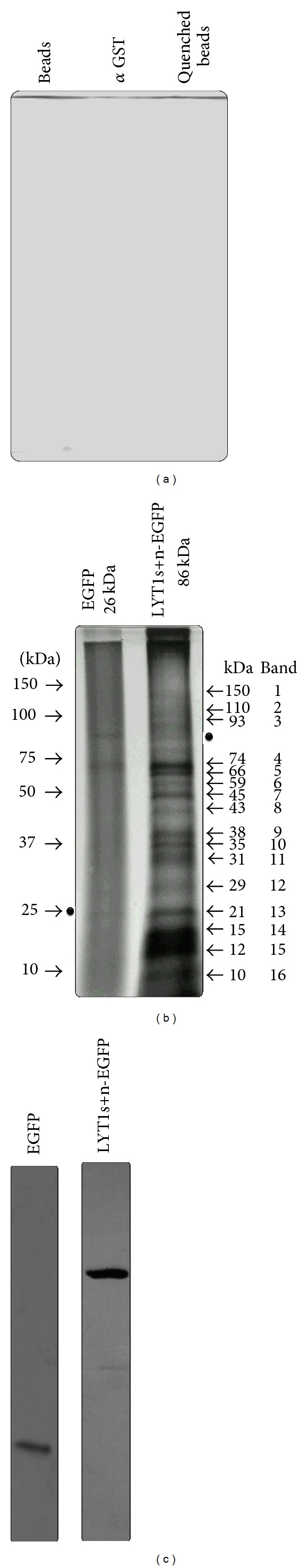
The LYT1 interaction profile. (a) Control gel (beads) composed of the same support material as the coimmunoprecipitation gel but not activated, a nonrelevant antibody (*α*-GST), and a quenched antibody coupling gel (quenched beads) were used as nonspecific interaction controls. (b) Protein extracts from EGFP and LYT1s+n-EGFP transgenic parasites were processed by immunoprecipitation with an *α*-EGFP antibody. Molecular weight markers (left of the gel), calculated molecular weights of the interaction products, and band numbers (to the right of the gel) are indicated. The positions of recombinant proteins are indicated with black spots. (c) A western blot containing the EGFP and LYT1s+n-EGFP coimmunoprecipitation products was probed with *α*-EGFP antibodies. Representative results of three independent experiments are shown.

To determine whether the LYT1 products interact with other proteins, coimmunoprecipitation assays were performed to establish the *in vivo* protein-protein interactions. Stably transfected parasites and the antibody against the EGFP tag were used for the assays as described in Section 2. As shown in Figure 3, the negative controls using only beads, an unrelated antibody (anti-GST) or quenched beads did not precipitate nonspecific products (Panel (a)). Moreover, as expected, in the control parasites expressing EGFP, we detected a protein of 26 kDa, the molecular weight of EGFP. There were also three other non-specific bands that were eliminated in the analysis for the tested samples (Panel (b)). This result allowed us to be certain that the conditions in which the columns and interaction solutions were prepared were adequate to perform the assays of the samples. When the LYT1s+n stable parasites lines were evaluated, approximately 16 bands ranging from 8 to 150 kDa were coimmunoprecipitated, suggesting that LYT1 interacts with different proteins (Panel (b)).

 Because LYT1 has lytic activity and is highly unstable, we determined whether the coimmunoprecipitation bands could be the result of the exogenous LYT1-EGFP protein degradation. Then, the coimmunoprecipitation products were analyzed by western blotting using an anti-EGFP antibody. As shown in Figure 3(c), a single band of approximately 83–86 kDa was observed in the LYT1s+n parasites, and a 26 kDa band was observed in the EGFP parasites, thus demonstrating the sample integrity and confirming the accuracy of the coimmunoprecipitation products.

### 3.4. Interaction Strength of the Coimmunoprecipitation Products

**Figure 4:**
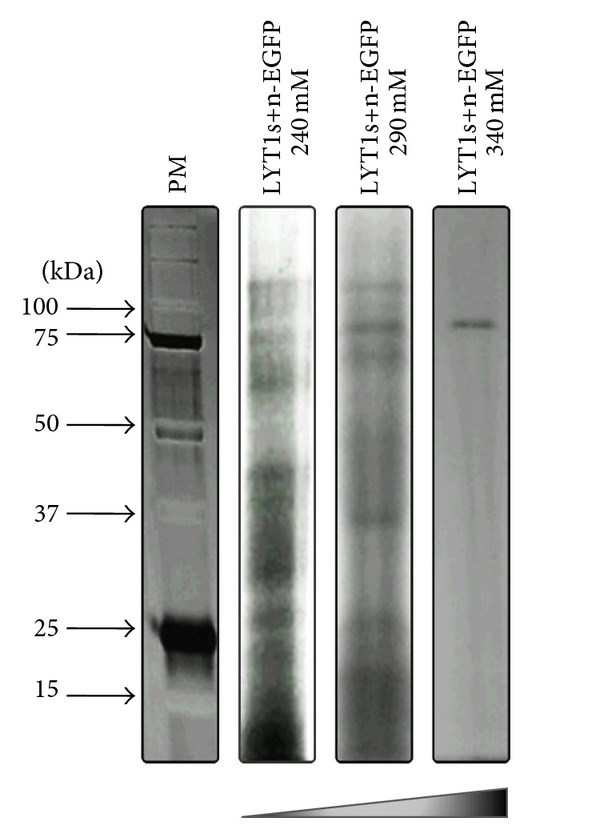
The interacting proteins bind to LYT1 with different affinity levels. To assess the protein-binding properties of the LYT1 interactome, increasing salt concentrations in the interaction wash buffer were used. Representative results of three independent experiments are shown.

Once the coimmunoprecipitation pattern of the LYT1s+n stably transfected parasites was determined, we evaluated the interaction strength of the proteins using increasing salt concentrations in the wash buffer. As shown in Figure 4 and Table 1, when 100 mM NaCl was used, the same coimmunoprecipitation pattern was observed. However, when the NaCl concentration was increased to 240 mM, 3 bands (of 66, 93 and 110 kDa) were lost, and 10 more bands (of 10, 12, 15, 21, 31, 35, 43, 45, 59 and 74 kDa) were lost when 290 mM NaCl was used. When the highest salt concentration was used (340 mM), only the 86 kDa band remained, which corresponds to the molecular weight of the exogenous LYT1 chimeric proteins.

 These results indicate that LYT1 binds to the various proteins that it interacts with at different affinities.

### 3.5. MS-MS Analysis of the Coimmunoprecipitation Products

To determine the identity of the LYT1 interaction profile, the coimmunoprecipitation products were analyzed by MS-MS (Q-TOF) tandem mass spectrometry and *in silico* analysis as described in Section 2. As shown in Table 2, we obtained 68 total peptides that corresponded to nine identified proteins: trans-sialidase (TS), kinetoplastid-specific DSPs (kDSPs), histone H2A and histone H2B, *α*- and *β*-tubulin, dynein, a tetratricopeptide repeat (TPR) protein (TcC31.24), LYT1, and five other hypothetical proteins with unknown function. The identified proteins are grouped into four functional groups: (1) infection process, (2) transcription, cell cycle, and development, (3) parasite motility and protein traffic and (4) interaction scaffold, which represent 13%, 25%, 19%, and 13% respectively. The remaining 30% corresponds to hypothetical proteins.

 These findings reveal that LYT1 interacts with different proteins, thus providing the first LYT1 interaction map for *T. cruzi*.

## 4. Discussion

 To increase the number of functions that a cell can carry out without increasing the number of genes, evolution has produced different solutions. For example, the cell can distribute the products of a single gene to more than one cellular compartment [6], and the proteins can carry out their functions within a complex network of protein interactions that enable them to act in concert [8].

 Eukaryotic cells are defined by the existence of subcellular compartments and organelles that contain specific proteins that allow them to regulate their cellular functions. Therefore, understanding how proteins interact is a significant area of study because it can provide insights into the differential functionality of proteins with dual localization.

 The dual targeting properties of LYT1 [4, 5] and its multifunctional capacity [2–4] provide an excellent model for the study of protein interaction networks, not only because the full and truncated LYT1 forms are distributed in different organelles [4], but also because the full form of LYT1 exhibits dual localization due to the presence of both a secretion signal and a nuclear localization signal [5]. Consequently, LYT1 products can be exposed to different microenvironments, and interactions with other proteins can modulate its function.

 We have identified some potential LYT1-binding proteins by coimmunoprecipitation assays using total protein extracts obtained from parasites that simultaneously express both forms of LYT1 fused to an EGFP tag. Here, the EGFP tag functions as prey, and the antibody against EGFP functions as bait. The advantage of this *in vivo* approach is that it was carried out under intracellular conditions, thereby enabling a better analysis of the physiological roles of LYT1 products. We identified LYT1-binding proteins that participate in the parasite infection process (TS), are related to transcription, cell cycle, and development (histone H2A and histone H2B and kDSPs), are involved in parasite motility and protein traffic (dynein and *α*- and *β*-tubulin), and that act as scaffolding proteins (TcC31.24 and kDSPs) that participate in the formation of multiprotein complexes involved in several aforementioned processes. Additionally, we also found five proteins that are annotated as hypothetical proteins or that have unknown function.

 Trans-sialidases (TS) are a family of membrane proteins that transfer sialic acid from the glycoconjugates of the host membrane to terminal beta-galactopyranosyl units present on the surface of the parasite and play a key role in the invasion of the mammalian host cell and immunomodulation of the infected host [17]. The critical role of TS in invasion has been highlighted the fact that invasion was neutralized by human antibodies again TS [18], TS+ parasites were highly invasive and more virulent than TS− or unfractionated parasites [19], and by the observation that high TS expression levels increase the exit of trypomastigotes from the parasitophorous vacuole and their subsequent differentiation into amastigotes [20]. With the results that we have produced so far, we do not know whether the interaction between TS and LYT1 is direct or via scaffolding molecules that are also found in the coimmunoprecipitation products (i.e., TcC31.24).

 TcC31.24 contains a TPR motif, which is one of the many repeated motifs that form structural domains in proteins that act as scaffolds in the formation of multiprotein complexes that are involved in numerous cellular processes, such as transcription, cell cycle, protein translocation, protein degradation, and host defense against invading pathogens [21]. As TcC31.24 participates in protein-protein interactions, it is not surprising that it is coimmunoprecipitated with TS, as both molecules are present in the parasite exosomal proteome [22].

 Exosomes, originally described in reticulocytes [23], are membrane vesicles that are released into the extracellular milieu by a variety of mammalian cells that play a role in antigen presentation, the transfer of MHC class I- and II-peptide complexes between cells of the immune system, T-cell stimulation, and membrane exchange among cells [24–26]. This type of vesicle has also been described in *T. cruzi*, in which they participate in the release of surface antigens [27]. A preliminary proteomic study of these vesicles reveals that they are rich in TS, gp63, tubulin, kinesin, dynein, HSP 70/90, and TPR hypothetical proteins, among other important proteins for *T. cruzi* virulence [22]. Therefore, TcC31.24 may function as scaffold protein between TS and LYT1, as both proteins are involved in the processes of parasite infection. As the exosomes contain several proteins with a common role in infectivity, we propose that the parasite can regulate the simultaneous secretion of all these proteins. However, future experiments will be necessary to demonstrate the presence of LYT1 in the exosomes and the coordinated secretion of functionally related proteins.

 Regarding kDSPs, sequence analysis showed that this protein may be localized to the cytoplasm or the membrane and that it contains the phosphatase conserved domain DSPC: dual specificity phosphatase catalytic domain. Protein phosphatases are conventionally classified according to their substrate preferences, including serine- and threonine-specific phosphatases (STP); tyrosine-specific phosphatases (PTP); dual-specificity phosphatases (DSP) that dephosphorylate phosphoserine, phosphothreonine, and phosphotyrosine substrates; lipid phosphatases (PTEN type and Myotubularins) and the low molecular weight PTP (LMW-PTP). The presence of specific conserved motifs in the catalytic domain as well as additional regulatory or targeting domains allow these types of protein phosphatases to be recognized and classified into different subfamilies [28–30]. Using these criteria, the TriTryp phosphatome has been recently reported, revealing that these organisms have an unusual composition of phosphatases, in which the PTP family is greatly reduced, whereas the STP family has expanded when compared with human phosphatases. Interestingly, a novel domain architecture was also identified in several phosphatases, and a number of atypical and unique phosphatases were found, suggesting potentially new pathways involving phosphatases [29]. The latter group contains the kDSPs family, which includes the putative phosphatase found in this work. The kDSPs are characterized by considerable divergence from classic DSPs in both their domain organization and sequence features. Although the kDSPs share most of the classic DSP motifs, they are significantly longer than human DSPs and can contain either N- or C-terminal extensions. Some of these extensions contain accessory motifs or domains, including the leucine-rich repeats (LRRs) that are present in scaffolding proteins in signaling pathways [31–33]. The extensions may also contain an ankyrin domain, which is a common protein-protein interaction domain found in proteins involved in transcription initiation, cell-cycle regulation, and signaling [34].

 It is well documented that highly specific protein kinases and protein phosphatases control a number of processes, including metabolic pathways, cell-cell communication, cell growth and proliferation, and gene transcription. Furthermore, mutational analysis demonstrated that these proteins have essential roles in the virulence and infection of pathogenic bacteria [35–37]. Although the specific roles of protein phosphatases in unicellular protists, in particular protozoan parasites such as *Trypanosomes* and *Leishmania*, are less well understood, recent work has identified several protein phosphatases and has highlighted the importance of these phosphatases in the regulation of essential developmental aspects of the life cycle of pathogenic kinetoplastids [38–43].

 These findings show a correlation between the functions of LYT1 and kDSPs with regard to the parasite infection and stage transition process, so that it is possible to imagine that the presence of both molecules in the coimmunoprecipitation product may have a functional meaning for these processes.

 Of the proteins that interact with LYT1 and that are related to transcription, the cell cycle, and development, our analysis detected the histones H2A and H2B. This finding is consistent with the LYT1 nuclear zone localization and the participation of this molecule in the parasite stage differentiation process as a negative regulator [2]. It is unlikely that LYT1 directly interacts with the histone because LYT1 does not contain domains normally associated with a histone-modifying role (e.g., acetyltransferase, methylase, and kinase). Therefore, one possible explanation of this result is the presence of the leucine zipper-like noncanonical domains found in the LYT1 sequence, which may allow its direct interaction with DNA and thus the coimmunoprecipitation of the DNA-binding histone. Another possibility is that LYT1 interacts with a histone-modifying molecule or molecules attached to it, through scaffolding proteins such as TcC31.24 and kDSPs, and that it could thereby potentially be associated with the nuclear membrane or telomeric regions. In this regard, it is interesting to note that the truncated form of LYT1 has a very similar pattern of localization [5] to that reported for *Leishmania major* telomeres, which are organized in clusters dispersed throughout the nucleus periphery in a speckled pattern [44].

 Unlike other trypanosomatids, in which a low proportion of their genomes is stage-regulated [45–47], *T. cruzi* displays stage-regulated control of mRNAs for more than 50% of its genes [48]. The apparent absence of typical promoters in *T. cruzi* has led to several findings that suggest that epigenetic mechanisms play a critical role in gene regulation in this parasite. Therefore, it is not surprising that through its interaction with histones, LYT1 may participate in the *T. cruzi *differentiation process although future experiments are necessary to evaluate such participation.

*T. cruzi*, as other trypanosomatid flagellate parasites, is characterized by the presence of a cytoskeleton that is responsible for maintaining cellular shape and its modulation among different life cycle stages [49]. Two of the most important cytoskeletal components are *α*-tubulin and *β*-tubulin, which polymerize into microtubules that form the parasite subpellicular corset and play an important role in the separation of the basal bodies [50] and the growth of the new flagellum as well as mitosis and cytokinesis [49–51]. The *α*-tubulin and *β*-tubulin association with LYT1 found in this work agrees with prior evidence that the secreted proteins, such as LYT1 [4], are exocytosed via the parasite flagellar pocket [52].

 The parasite flagellar pocket is a structure formed primarily by microtubules [53] and together with the cytostome, is involved in the *T. cruzi *endocytic and exocytic pathways [53–56]. In these pathways microtubules function as roadways for mechanochemical motor proteins such as kinesin and dynein, using the energy of ATP hydrolysis to transport membrane-bound organelles as well as other structures within the cell, and it has been implicated in vesicular transport to and from the Golgi complex [57]. 

Kinesin and cytoplasmic dynein have been recognized as the two main microtubule-associated motors, with kinesin involved in the plus-end-directed transport and dynein in the minus-end-directed transport [58]. Therefore, the association of LYT1 with microtubules could result from its interaction with dynein during its transportation to the membrane.

 These findings suggest that the association of LYT1 with *α*-tubulin, *β*-tubulin, and dynein may more likely result from the LYT1 secretion system rather than an interaction that is necessary for LYT1's function.

 Finally, we also found LYT1 peptides in the interaction profile. The presence of these peptides is expected because exogenous LYT1 was used as bait in the coimmunoprecipitation assays. However, the presence of coiled coil sequences in LYT1 supports the possibility that this protein interacts with itself to produce multimers with pore-forming activity. Several intracellular pathogens, including bacteria and protozoa, produce pore-forming proteins (PFPs) to escape from phagosomes and thereby survive within the cell [59–63]. Tc-TOX, a protein functionally related to LYT1, is secreted into the acidic environment of the phagosome, possibly by forming pores in the membrane which contribute to the parasitophorous vacuole disruption [64]; LYT1 shares many molecular and functional characteristics [2–4] with TcTOX [64], for example, both are secreted proteins, are recognized by anti-C9 antibodies, and show hemolytic activity at low pH. Therefore, we believe that it is important to evaluate the possible ability of LYT1 to function as a PFP. 

 Future experiments also will be necessary to determine the function of proteins classified as hypothetical or with unknown function or homology and to validate the biological significance of the LYT1 interaction with them.

This proteomic approach provides the identification of putative partners of LYT1 from *T. cruzi*. Eventually, these newly identified proteins should be analyzed to complement this *in vivo* study in order to help understand the mechanism involved in not only LYT1 multifunctionality but also the molecular pathogenesis of *T. cruzi* infection and to develop novel approaches of intervention in Chagas disease.

## Figures and Tables

**Table 1:** Interaction strength of coimmunoprecipitation products.

Co IP products (kDa)	240 mM (kDa)	290 mM (kDa)	340 mM (kDa)
150	150	150	
110			
93			
86	86	86	86
74	74		
66			
59	59		
45	45		
43	43		
38	38	38	
35	35		
31	31		
29	29	29	
21	21		
15	15		
12	12		
10	10		

The molecular weights of the LYT1 co-immunoprecipitation products (from Figure 3) and under the treatment with increasing salt concentration (240 mM, 290 mN, and 340 mM) in the wash buffer (from Figure 4) were calculated with respect to the molecular weights of standard proteins as described under “Section 2.”

**Table 2:** Proteins identified by MS-MS (Q-TOF) tandem mass spectrometry.

Group	Theoretical MW (kDa)	Protein name	Accession number (TriTrypDB)	Number of identified peptides	Sequence coverage (%)
Infection process (13%)	59	LYT1p	TcCLB.508045.40	4	12%
	85	Trans-sialidase, putative	TcCLB.506331.90	6	8%
Transcription, cell cycle, and development (25%)	13	Histone H2B	Tc00.1047053511635.10	7	21%
	59	LYT1p	TcCLB.508045.40	4	12%
	35	Dual specificity protein phosphatase	TcCLB.504741.170	5	9%
	15	Histone H2A	Tc00.1047053511817.151	5	8%
Parasite motility and protein traffic (19%)	49	*β*-Tubulin	Tc00.1047053411235.9	4	10%
	50	*α*-Tubulin, putative	Tc00.1047053411235.9	7	8%
	282	Dynein heavy chain, putative	TcCLB.508815.179	4	3%
Interaction scaffold proteins (13%)	35	Dual specificity protein phosphatase	TcCLB.504741.170	5	9%
	80	TcC31.24	Tc00.1047053506529.460	4	7%
Hypothetical proteins (30%)	70	Hypothetical protein, conserved	TcCLB.506755.10	8	6%
	35	Hypothetical protein, conserved	TcCLB.511301.50	2	5%
	85	Hypothetical protein, conserved	TcCLB.509341.20	2	4%
	90	Hypothetical protein, conserved	TcCLB.506529.460	4	3%
	255	Hypothetical protein, conserved	TcCLB.511511.10	6	2%
